# Development and Validation of MPS-Based System for Human Appearance Prediction in Challenging Forensic Samples

**DOI:** 10.3390/genes13101688

**Published:** 2022-09-21

**Authors:** Filomena Melchionda, Beatrice Silvestrini, Carlo Robino, Carla Bini, Paolo Fattorini, Cristina Martinez-Labarga, Flavio De Angelis, Adriano Tagliabracci, Chiara Turchi

**Affiliations:** 1 Section of Legal Medicine, Department of Biomedical Sciences and Public Health, Polytechnic University of Marche, Torrette, 60126 Ancona, Italy; 2 Department of Surgical, Medical, Molecular Pathology, and Critical Area, University of Pisa, 56126 Pisa, Italy; 3 Department of Public Health Sciences and Pediatrics, University of Turin, 10126 Turin, Italy; 4 S.C. Medicina Legale, AOU Città della Salute e della Scienza, 10126 Turin, Italy; 5 Department of Medical and Surgical Sciences, Section of Legal Medicine, University of Bologna, 40126 Bologna, Italy; 6 Department of Medicine, Surgery and Health, University of Trieste, Strada di Fiume 447, 34149 Trieste, Italy; 7 Centre of Molecular Anthropology for Ancient DNA Studies, Department of Biology, University of Rome “Tor Vergata”, 00173 Rome, Italy

**Keywords:** forensic DNA phenotyping, MPS, degraded DNA, HIrisPlex-S system, externally visible trait

## Abstract

Forensic DNA phenotyping (FDP) provides the ability to predict the human external traits from unknown sample donors, directly from minute amounts of DNA found at the crime scene. We developed a MPS multiplex assay, with the aim of genotyping all 41 DNA markers included in the HIrisPlex-S system for simultaneous prediction of eye, hair and skin colours. Forensic samples such as blood, skeletal remains, touch DNA, saliva swab, artificially degraded samples together with individuals with known phenotypes and a set of 2800 M control DNA were sequenced on the Ion Torrent platform in order to evaluate the concordance testing results and the forensic suitability of the 41-plex MPS assay. The panel was evaluated by testing a different number of PCR cycles and the volume of reagents for library preparation. The study demonstrated that full and reliable profiles were obtained with 0.1–5 ng, even with high degraded DNA. The increment of the number of PCR cycles results in an improvement of correctly genotyping and phenotyping for samples with low amounts of degraded DNA but higher frequencies of artefacts were found. The high DNA degradation level did not influence the correct genotyping and phenotyping and the critical parameter affecting the result is the quantity of input DNA. Eye and hair colour was predicted in 92.60% of individuals and skin colour in 85.15% of individuals. The results suggest that this MPS assay is robust, highly sensitive and useful for human pigmentation prediction in the forensic genetic field.

## 1. Introduction

Forensic DNA phenotyping (FDP) is a set of innovative genetic tools that allows the age, appearance and biogeographical ancestry (BGA) prediction of unknown perpetrators from various biological traces found at the crime scene. In recent years, this new branch of forensic genetics has gained a great deal of importance for its potential applications as a supplementary investigative tool whenever conventional DNA profiling fails to provide a match with any reference profiles or genetic profiles entered in criminal databases. The FDP outcomes are provided in terms of “prediction”, i.e., are probabilistic, and they can only infer a specific phenotypic feature to a certain degree of probability (probabilistic inference). It must be kept in mind that FDP is not an identification tool but rather an investigative tool that helps identify unknown suspects or to help with non-criminal missing person cases. DNA phenotyping is indicated as a “biological witness” because it acts as a witness that describes the appearance of a person of interest, potentially providing even more accurate information than the human eyewitness does, which is known to be unreliable. 

One branch of the FDP tool, which is particularly advanced and studied, is the prediction of the appearance, i.e., the externally visible characteristics (EVCs); both because these phenotypic characteristics are determined by a relatively low number of genes, making these the least complex genetic trait to analyse [1] and because of their high heritability, which suggests how genetic results can be easily predicted. 

Currently, the 41 DNA polymorphisms of the HIrisPlex-S system [2] represent the most complete DNA-based prediction tool for the simultaneous prediction of eye, hair and skin colour. This predictive method reflects an extension of the previously developed IrisPlex system [3] that allowed only eye colour prediction based on six DNA variants and the HIrisPlex for simultaneous eye and hair colour predictions consisting of 24 DNA variants [4]. All these methods were based on SNaPshot single base extension technology and capillary electrophoresis (CE). However, the SNaPshot genotyping test has several restrictions; the main limitation was related to the limited number of DNA variants that can be evaluated per single test, which leads to running multiple assays with cost and time increases. As consequence, more assays mean more consumption of DNA that in some cases may not always be available. This limit can be overcome using massively parallel sequencing (MPS) technology, which provides new opportunities to obtain genetic data for hundreds of SNP loci in a single assay, even at low levels of input DNA [5,6]. Recently, more researchers have started to apply massively parallel sequencing technologies for ECVs application for both commercial [7,8,9] and non-commercial developments [10]. However, only a few MPS studies concerning forensic DNA phenotyping focused on degraded DNA samples, i.e., DNA samples with poor quality or low quantity of DNA [9,10,11,12,13,14] whose analysis often shows partial or inconclusive genetic results.

Most of these studies [9,10,11,12,14] reported the performance of the MPS assay on a set of forensic samples, different for biological tissue, and artificially degraded samples obtained with sonication treatment. The results showed well-balanced tests suitable for forensic casework by their high sensitivity with full SNP profiles obtained down to 100 pg of DNA input. However, more challenging and naturally degraded real casework samples should be studied. Kukla-Bartoszek et al. reported a preliminary validation of the MPS test [13], based on the Ion AmpliSeq™ HIrisPlex-S panel using Ion Torrent technology [10]. The authors performed the analysis on sixty-three bones, that showed different levels of DNA degradation, and their results highlighted the ability of this assay to yield full and reliable profiles using up to 50 pg of low and degraded DNA samples. Furthermore, the authors did not find a significant correlation between DNA degradation and phenotyping success.

In the present study, we describe the development and validation of an MPS multiplex assay, based on Ion Torrent technology, targeting all the 41 SNPs included in HIrisPlex-S system. The MPS panel was designed to maintain the size of the amplicons below 180 bp, in order to investigate the usefulness of the panel with a broad kind of forensic casework sample, including different biological matrices and containing different levels of DNA quantity and quality. 

## 2. Material and Methods

### 2.1. DNA Samples

To investigate the effectiveness of the designed panel, a selection of forensic samples (Supplementary Table S1) consisting of different biological matrices, were collected by five laboratories. The test samples included buccal swabs of individuals with known phenotype (*n* = 5), touch DNAs (*n* = 5) together buccal swab (*n* = 5) of same donor used as reference, blood samples (*n* = 5), skeletal remains (*n* = 9, of which five bones and four teeth) and artificially degraded DNAs (*n* = 2), produced with a method based on aqueous hydrolysis of the DNA in vitro following the published protocols in [15]. Regarding the skeletal remains, a part consists of archaeological remains provided by the Centre of Molecular Anthropology for Ancient DNA studies (Department of Biology, University of Rome Tor Vergata), following the permissions by the Anthropology Service, Soprintendenza Speciale Archeologia, Belle Arti e Paesaggio di Roma. They belong to a Roman Imperial (1st–3rd cent. CE) cohort from the Rome metropolitan area, which was increasingly analysed over the years [16,17,18,19,20,21,22,23]. Teeth (CM132 and AP1), petrous (BAS539), or tubular bones (VAL2, FIDENE, BASL3) were selected for sampling according to district availability. 

The samples were processed in the Centre of Molecular Anthropology for Ancient DNA studies, University of Rome Tor Vergata (00173 Rome, Italy), set specifically for ancient DNA recovery and processing, whose access is strictly restricted. The samples were UV-irradiated at 6 J/cm^2^ 254 nm for 12 h. The powder for DNA extraction was produced using a Dremel drill applying the lowest speed and collected into vials. The weight of the obtained powder was 0.1 g. The powder was incubated rotating for 24 to 48 h at 37 °C in 1 mL of extraction buffer—Urea in EDTA 0.5 M and 10 µL of Proteinase K 20 mg/mL [24] and negative control was set and maintained through the extraction protocol. The supernatant was collected and transferred to Amicon Ultra-4 Centrifugal Filter Unit (Millipore, Burlington, MA, USA) with Ultracel-30 from Millipore for spinning down to 100 mL. DNA was extracted and purified using MinElute spin columns and Qiagen buffer (Qiagen, Hilden, Germany) and was stored at −20 °C. 

The Centre of Molecular Anthropology for Ancient DNA studies (Department of Biology, University of Rome Tor Vergata) provided also three blood samples consisting of DNA from an Amhara donor from Asela, Oromia Region, central Ethiopia (AM11) [25]; one from a donor from the Ecuadorian community of African ancestry living along the Rio Cayapas in the Esmeraldas province (RC544) [26,27,28,29]; and 1 sample from China (PK48) [30]. For these samples, genomic DNA was isolated from whole blood through the salting out method [31] and the extracted DNA was stored at −20 °C.

For the other selected samples, different DNA extraction and quantification methods were performed. The details of the tested samples are given in Supplementary Table S1 [30,31,32,33,34,35,36,37].

Sensitivity study was performed by using the 2800 M (Promega, Madison, WI, USA) as control DNA, diluted to concentrations of 5 ng/µL, 1 ng/µL, 500 pg/µL, 100 pg/µL, 50 pg/µL and 25 pg/µL.

### 2.2. Assay Development, Library Preparation and MPS Sequencing 

The 41 SNPs of HirisPlex-S systems [2] were analysed in this study. The library PCR primer pairs were designed with the Ion AmpliSeq Designer tool (TFS, https://ampliseq.com/ (accessed on 7 March 2019)), using the FFPE DNA type option (125–175 bp amplicon range) in order to allow analysis of degraded DNA. Two primer pools were designed to amplify 35 amplicons covering the 41 SNPs.

The MPS assay design was evaluated by testing different numbers of PCR cycles (21, 23 and 25) and different reagents volume were used in the library preparation steps. Forty-eight libraries were sequenced in this study, seven of which were prepared in half volume test, i.e., the volume of all reagents used, and the library building was half of that recommended in the protocol. The amount of input DNA, number of PCR cycles and other information about MPS libraries are reported in Supplementary Table S2.

The MPS libraries were prepared using the Precision ID Library Kit (TFS) according to Ion AmpliSeq DNA and RNA library preparation user guide (MAN0006735, Rev C.0). The amplification of each primer pool was performed in 10 μL PCR reactions and combined after target amplification to yield a total volume of 20 μL. Thermal cycling was performed on the Veriti™ 96-Well Thermal Cycler (Applied Biosystems, Waltham, MA, USA; TFS) using the following conditions: enzyme activation for 2 min at 99 °C, followed by 99 °C for 15 s and 60 °C for 4 min. After PCR reactions, 2 μL FuPa Reagent was added to partially digest primer sequences (TFS) and incubated for 10 min at 50 °C, for 10 min at 55 °C, for 20 min at 60 °C. An amount of 4 µL Switch Solution, 2 µL DNA Ligase, 2 µL diluted Ion Xpress™ Barcode Adapters were added into 22 µL digested PCR reaction for ligation of libraries with adaptors, and the mix was then incubated for 30 min at 22 °C, 5 min at 68 °C, 5 min at 72 °C. Then the libraries were purified with Agencourt™ AMPure™ XP Reagent (Beckman Coulter, Brea, CA, USA) and the final concentration of each barcoded library (Supplementary Table S2) was determined using the TaqMan^TM^ Library Quantification kit (TFS, Waltham, MA, USA) on a Rotor-Gene Corbett 6000 (Qiagen) following the manufacturer recommendations. 

Two different Ion Torrent platforms, Ion Personal Genome Machine™ (PGM™) System and Ion Gene Studio S5^TM^ System (TFS) were used for sequencing the forty-eight libraries of the present study. Thirty-eight barcoded libraries were diluted to 100 pM, pooled in equal volume aliquots and then submitted to emulsion PCR to generate template positive Ion Sphere™ Particle (ISPs) containing clonally amplified DNA. Emulsion PCR (emPCR) was performed in the Ion OneTouch™ 2 Instrument (TFS, Waltham, MA, USA) with the Ion PGM™ Hi- Q™ View OT2 Kit (TFS, Waltham, MA, USA). The template-positive ISPs were enriched on Ion OneTouch™ ES (TFS) and sequenced on Ion Personal Genome Machine™ (PGM™) System by using Ion PGM™ Hi- Q™ Sequencing Kit (TFS, Waltham, MA, USA), two Ion 318™ Chip v2 (TFS, Waltham, MA, USA) types and 200 read mode. The remaining ten libraries were sequenced on Ion Gene Studio S5^TM^ (TFS, Waltham, MA, USA), performing the libraries enrichment and chip loading steps using the Ion Chef™ (TFS, Waltham, MA, USA) and the Ion 510™ and Ion 520™ and Ion 530™ Kit (TFS, Waltham, MA, USA). An appropriate volume of each manually prepared library, adjusted to 30 pM concentrations, was combined to create an approximately equimolar pooled library for the Ion Chef™ (TFS, Waltham, MA, USA) and it was loaded on Ion 520 Chip. 

### 2.3. Sequencing Data Analysis

All raw data were processed by the Torrent Suite (v. 5.0.4/5.10.1) and the reads were aligned against human reference genome (GRCh37/hg19). Coverage analysis was carried out by Coverage Analysis (v.5.0.4.0/v.5.10.0.3) plugin, which provided information about mapped reads, on-target percentage and mean depth of coverage downloadable for each sample library (Supplementary Table S2). Sequencing reads for each library were analysed through HID SNP Genotyper Plugin (v.4.3.2) with default settings as: minimum allele frequency = 0.1, minimum coverage = 6, minimum coverage on either strand = 0, or maximum strand bias = 1. This updated version of the HID SNP Genotyper has several QC filters that identify potentially incorrect genotype calls. The software Integrative Genomics Viewer (IGV, v.2.8.0, Broad Institute and UC San Diego) [38] was used to verify the call variants flagged by QC filters. PMDtools [39] was used to compute ancient DNA damage patterns and to identify degraded sequences that were likely to be endogenous. Each read from the bam file was assigned a PMD score, and PMD scores > 3 allow for filtering ancient DNA molecules from the others. For final interpretation, the threshold for the “locus call” (i.e., its genotyping) was fixed at 50×, suggested as reliable threshold for forensic challenging samples [40], comprising a minimum allele frequency for heterozygote calling of 0.1. 

Certainly, the applied threshold for PMDtool should be considered stringent, preventing obtaining the 50× coverage aimed to obtain a proper “locus call”. Indeed, this value is hard to obtain, whichever is the ancient DNA sample, as the DNA preservation for archaeological specimens is usually mined by taphonomy and the chemical–physical processes occurring in the deposition environment, which is detrimental to the proper preservation of ancient DNA [41]. So far, previous analyses of ancient Romans from Rome pointed out similar preservation issues [22,23,42]. Accordingly, as the use of the PMD scores shrinks severely the number of available reads making the inference unattainable, our phenotyping estimates for the ancient samples will be definitely accomplished using the unfiltered bam files, even recognizing that these interpretations should be considered cautiously, even accounting for the archaeological background of the samples.

For performance evaluation of the designed panel, the relative depth of coverage (rDoC) across all target sequences was calculated as the ratio of depth of coverage (DoC) at single amplicon to total DoC of the sample.

### 2.4. HIrisPlex-S Model Tool and Guide

Genotype data of the 41 DNA variants can be uploaded to the easy-to-use DNA Phenotyping web tool found at https://hirisplex.erasmusmc.nl/ (accessed on 15 April 2022), to generate individual predictions of eye, hair and skin colours. Notably, this software is able to generate additional predictions of hair colour shades (light/dark). The model is based on 9466 individuals for eye colour, 1878 and 854 individuals for hair colour and hair colour shades (light/dark) prediction, respectively, and lastly 1423 for skin colour. Prediction results were reported as predictive probability values (*p*-values) for each category. The eye colour prediction result consists of three categories: blue, intermediate and brown; the highest *p*-value was considered as the predicted eye colour. This tool provides a high discrimination level between blue and brown colour categories, but accuracy is lower for intermediate colour prediction. The hair colour prediction result consists of four categories: blond, brown, red and black. Finally, the skin colour prediction result consists of five categories, based on a dermatological established Fitzpatrick scale [43] for skin colour and sun sensitivity, as very pale, pale, intermediate, dark and dark to Black. For hair and skin colour predictions the highest probability category approach in combination with the recommended prediction guide approach [2,44], which also takes shade into account for hair colours, was evaluated. The accuracy of prediction performance by the HIrisPlex webtool was evaluated by calculating AUC (area under the receiver operating characteristic curve) for each category. 

## 3. Results and Discussion

### 3.1. Sequencing Data Overview

The main parameter of the two Ion 318™ Chip v2 and Ion 520 Chip run in this study are reported in Supplementary Table S3. In summary, for the forty-three libraries loading into two Ion 318™ Chip v2, on 11,287,275 addressable wells, 72.3% and 67.6% showed Ion Sphere™ Particles (ISPs) on average, with more than 99% of the ISPs represented by the libraries. The final ISPs libraries were 4,705,047 and 4,244,767 (58.5% and 56.4% of the total), respectively, for first and second chip. The percentage of low quality was 10.0% and 8.5% and the one of adapter dimer sequences was 4.4% and 4.9%, respectively, for the first and the second Ion 318™ chip. Conversely, for the library sequenced into Ion 520 Chip, on 12,530,194 addressable wells, 95% showed Ion Sphere™ Particles (ISPs) on average, with more than 99% of the ISPs represented by the libraries. The final ISPs libraries were 6,142,668 (52% of the total). The percentage of low quality and adapter dimer sequences was very low (4.4% and 1.4%, respectively).

### 3.2. Relative Depth of Coverage/Coverage Analysis

The 41 DNA markers for eye, hair and skin colour prediction were covered by 35 amplicons, given the short distance of 8 SNPs on the *MC1R* gene. The amplicons in this panel were designed to be relatively short (size ranging between 124–174 bp), which are especially advantageous for the analysis of challenging forensic samples. The MPS results (Supplementary Table S2) showed a very good performance of the designed panel. The average coverage of sequenced libraries by MPS was 3833.15 and the uniformity of coverage of 92% (mean). In the half-volume tests the concentrations of the libraries, amplified with the same DNA input and PCR cycles, were approximately the same as in the libraries performed with the recommended volumes. 

To evaluate the performance of the designed panel the relative depth of coverage (rDoC), which is the ratio between the coverage of each locus and the overall coverage of the sample, was assessed separately for reference samples and degraded DNA samples across all loci. A uniform distribution in all 41 DNA markers both in reference samples and in degraded DNA samples, regardless of the amplicon size, was found. 

**Figure 1:**
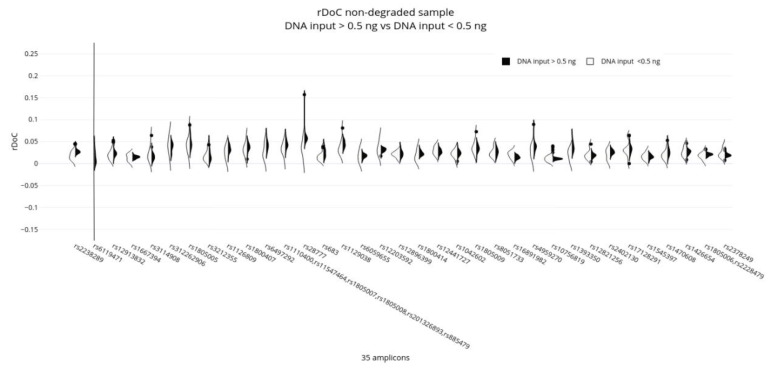
Comparison of rDoC distribution observed in non-degraded samples pooled in two groups according to the DNA input used for amplification, named as follows: DNA input > 0.5 ng and DNA input < 0.5 ng. *X*-axis: polymorphisms included in the amplicons; *Y*-axis: value relative depth of coverage (rDoC).

To evaluate if the input DNA amount can affect the panel performance, the rDoC for non-degraded DNA samples (i.e., reference samples) was evaluated after pooling the samples in two groups according to the DNA input used for amplification, named as follows: DNA input > 0.5 ng and DNA input < 0.5 ng. All loci displayed a good uniformity regardless of amplicon length and DNA input used for amplification (Figure 1), except one amplicon (AMPL7160226302, rs6119471) which displayed abnormal data in samples with DNA input < 0.5 ng. 

**Figure 2:**
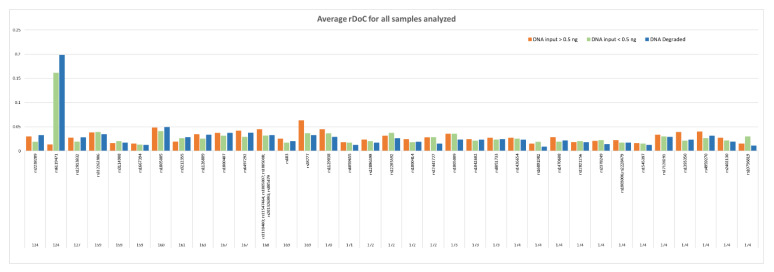
Comparison of average rDoC distribution observed in non-degraded samples (DNA input > 0.5 ng and DNA input < 0.5 ng) and DNA degraded samples. *X*-axis: mean relative depth of coverage (rDoC) value; *Y*-axis: polymorphisms included in the amplicons together with the amplicon size.

The trend of rDoC distribution in degraded samples remains quite homogeneous in all loci (Figure 2), except the AMPL7160226302, which displayed a remarkable increase in rDoC value, as previously observed in samples with DNA input < 0.5 ng. 

**Figure 3:**
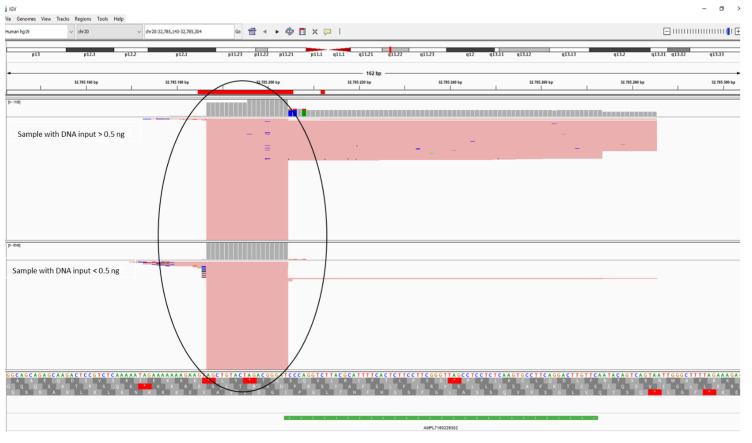
Visualization of sequence alignment to hg19 for AMPL7160226302 (rs6119471) with IGV software. In the circle, the dimers present in the samples.

To understand the reasons for the irregular rDoC values shown by the amplicon AMPL7160226302, an in-depth analysis of sequencing data was conducted by Integrative Genome Viewer (IGV) software (Figure 3) comparing two non-degraded samples, one amplified with DNA input greater than 0.5 ng and the other amplified with DNA input less than 0.5 ng. We noted that there are a high number of short reads in both sets of reference samples, even if this phenomenon is more accentuated in the samples with DNA input lower than 0.5 ng as mentioned before. We assumed that these reads probably represent dimers not filtered by the Coverage Analysis plugin and aligned to the human reference genome (GRCh37/hg19). The alignment was due to an overlap between the 3’ nucleotide position of the dimers and the first nucleotide of the target region, defined by the panel-designed bed file. These dimers lead to higher coverage in amplified samples with a low amount of DNA, but few are the reads that cover the polymorphism of interest, not allowing for a genotypic call in some samples.

### 3.3. HID SNP Genotyper Plugin

The generated data were processed with HID SNP Genotyper (v.4.3.2, TFS, Waltham, MA, USA) a software plugin that analyses barcoded samples, and then finds the genotype at positions specified in the bed file. The plugin applies a locus-level quality checks filter to flag possible incorrect data due to one of the following: coverage (COV) is less than twice the standard deviation compared to the mean; percentage positive coverage (PPC), the ratio of coverage from positive strand to negative strand is <30% or >70%; major allele frequency (MAF) ratio of major allele coverage to total coverage is <35% or >65% for heterozygotes and >95% for homozygotes; genotype not valid (NOC) observed when an “NN” genotype is called. It must be highlighted that a flag does not necessarily mean that there is an error but that a detailed revision of that call is required [45]. Furthermore, secondary data analysis has been processed through Integrative Genomics Viewer (IGV) software to check the call variants flagged by QC filters. Based on the type of samples analysed, we decided to consider the 50× coverage threshold for genotype calls as identified in [40]; so, for all the polymorphisms that showed a coverage lower than 50 reads, no genotype was assigned (NN). A total of 170 flags were scored in the forensic samples; most consisting of PPC flags (39.41%) followed by MAF flags (31.18%), NOC flags (20.59%) and COV flags (8.82%). In all cases, the in-depth analysis performed with IGV software confirmed the genotypes detected by HID SNP Genotyper plugin. The genotypes of the samples tested and uploaded to HIrisPlex-S Webtool take into account the loci flagged by the plugin but which, at the IGV manual inspection, showed a correct allelic call.

### 3.4. Sensitivity Study

**Figure 4:**
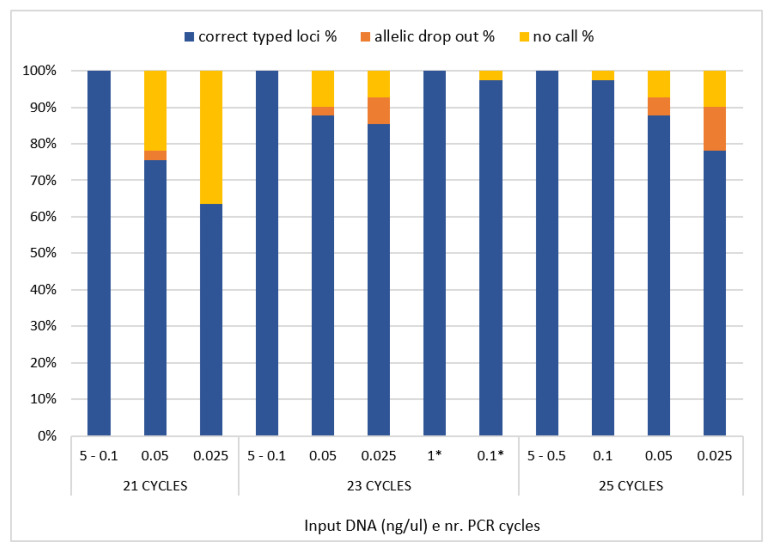
The figure shows the assessment of 41-SNPs panel sensitivity on control DNA samples. Reliable profiles were observed with 100 pg DNA input or higher. An increase in allelic drop-out events and decrease in no-call were observed with lower amount of DNA. * Libraries prepared with half reaction volume.

The decreasing amount of 2800 M Control DNA (Promega), ranging from 5 ng to 25 pg, was used as template DNA to test the sensitivity of the designed panel. The scalar amounts of control DNA were amplified at 21, 23 and 25 PCR cycles and, as expected, a reduction in reading depth was observed with the decreasing DNA input. The HID SNP Genotyper plugin (v.4.3.2) was used for the allelic caller and results showed that complete and reliable genotypes could be obtained with an amount of DNA up to 0.1 ng at 21 and 23 PCR cycles (Figure 4). When using 50 pg of input DNA and 21 cycles, 76% of correct genotypes were observed (Figure 4), with one allelic drop-out event (rs1129038). The same quantity of input DNA amplified with 23 PCR cycles results in 88% of correctly genotyped loci, one allelic drop-out event (rs1667394), and four no-call genotypes (rs16891982, rs4959270, rs1393350, rs6119471). Meanwhile, when 25 PCR cycles were applied, we observed 90% of correct genotypes, a decrease in no-call genotypes (rs1129038 and rs6119471), but an increase in allelic drop-out events (rs3114908 and rs1667394). Finally, when 25 pg of DNA was amplified at 21 PCR cycles 63% of correct genotypes were observed, with the remaining loci not called; with 23 and 25 PCR cycles we observed 85% and 78% of correct genotypes, respectively, and three (rs12821256, rs12913832 and rs683) and four (rs12821256, rs12913832, rs683 and rs8051733) allelic drop-out events, respectively (Figure 4).

For the libraries prepared with a half reaction volume, the results are consistent with those prepared with a standard volume, except for one no-call in samples with 0.1 ng. However, the no-call involved the amplicon AMPL7160226302, which showed in this assay critical results with less than 100 pg of DNA. Based on these results, for this panel we used in the remaining samples 23 PCR cycles, with the sensitivity threshold set to 100 pg. The prediction of pigmentation traits of 2800 M Control DNA (Promega) was performed using the prediction guide recommendations [2,44,46] and describes an individual with brown eyes (*p*-value 0.836), dark brown/black hair (*p*-value 0.521 for brown category and *p*-value: 0.742 for light shade) and lighter intermediate skin (*p*-value: 0.774 for intermediate category affected by pale category with *p*-value: 0.201). 

### 3.5. Genotyping Data and Phenotype Predictions with HIrisPlex-S Web Tool

All DNA samples were subjected to MPS sequencing using the Ion Torrent technology. The phenotype prediction was performed using the HIrisPlex-S web tool, which allows a simultaneous prediction of eye, hair and skin colour. The appearance prediction was easily obtained for the samples showing complete genotyping profiles, while different considerations should be made for the interpretation of the remaining 26.92% of partial profiles. Notably, the prediction accuracy in case of partial profile is strongly affected by missing genotyped markers and the lack of data could be detrimental to the final prediction, resulting in the loss of AUC (area under the curve, the measure of the ability of a classifier to distinguish between classes) [46].

The HIrisPlex-S system does not exclude the possibility of predicting eye, hair or skin colour for partial profile, unless the missing genotypes are all *MC1R* variants for hair colour prediction and the *HERC2-SLC45A2-IRF4* gene set for eye and hair colour prediction [46]. 

#### 3.5.1. Results of Known Phenotype Samples

Five buccal samples of individuals with known phenotypes were analysed to test all predictable categories for eye, hair and skin colours obtainable by HIriSplex-S system webtool. The five individuals with known phenotypes were amplified with 5 ng of DNA input and using 25 PCR cycles. The mean amplicons coverage values observed in these samples ranged between 2451 and 6499 (Supplementary Table S2). The coverage (COV) flag at rs6119471 was present in three samples but after the IGV check, the genotypes assigned by the HID SNP Genotyper plugin were confirmed. The predicted phenotypes agree with the expected ones; however, discrepancies in skin colour were obtained for two samples. In one case, an expected very pale skin failed to be predicted as the results showed a *p*-value of 0.393 for pale skin and for 0.544 for intermediate skin. Likewise, for the sample FG, we expected a dark skin colour but the webtool predicted a dark to Black category (*p*-value 0.962). However, these discrepancies in skin colour could depend on the subjective judgment of the researcher who recorded the phenotypic categories of the individual samples at the time of collection. The phenotype prediction is reported in Supplementary Table S4. 

#### 3.5.2. Results of Artificially Degraded DNA

In the present study, we evaluated two artificially degraded DNA together with their non-degraded sample reference. One sample (TS29) with high degraded DI (DI = nc; not calculable) was amplified with approximately 1 ng of input DNA using 21 PCR cycles, as well as its reference sample (TS26). A full genotype concordant with the reference sample profile was obtained. The subject presents blue eyes, blond/dark blond hair and pale skin. The other artificially degraded DNA sample (TS22) which showed DI not calculable and a low amount of DNA (not quantifiable as showed DNA quantity below the limit of quantification, loq) was amplified using 21 PCR cycles. Partial profiles were obtained with only a few loci correctly genotyped (6/41 SNPs) but with coverage lower than genotyping threshold fixed and therefore no predictions were possible. These results obtained by artificially degraded samples were very relevant, confirming that the high degradation index did not influence the correct genotyping and the critical parameter that affects the result is the quantity of DNA input. 

#### 3.5.3. Results of Modern Population Samples

In this study, three blood samples from three different modern populations were evaluated. The sample named RC544 belongs to a donor from the Ecuadorian community of African ancestry living along the Rio Cayapas in the Esmeraldas province [26,27,28,29]; the second, AM11, belongs to an Amhara donor from Asela, Oromia Region, Central Ethiopia [25], and the last sample, PK48, belongs to a subject from China [30].

The maximum amount allowed by the protocol, in terms of ul, was amplified using 23 PCR cycles because the samples showed inhibitors and a low amount of DNA upon quantification. In detail, the samples AM11 and RC544 were amplified with 1.8 ng and 4 ng of input DNA, respectively, using 23 PCR cycles, while the PK48 sample was amplified with 0.1 ng of input DNA. The coverage obtained for these samples reflected the amount of DNA used in PCR reactions; indeed, the coverage of PK48 samples was 438.3, and unlike the other two samples, had only 42% reads on target and a partial profile was obtained (38/41 SNPs). Despite the lack of three SNPs (rs17128291, rs6119471, rs3212355), the HIrisPlex-S webtool allowed a complete phenotype prediction which for this sample showed an individual with brown eyes, dark brown/black hair and intermediate skin as reported in Supplementary Table S4. Note that the three missing polymorphisms are involved in the regulation of skin pigmentation but do not have such an impact as to modify the final phenotypic prediction. The other two samples displayed full profiles with a phenotypic prediction of subjects with brown eyes, black hair and dark to Black skin (Supplementary Table S4). The skin prediction of RC544 agrees with that reported in a previous study [47] in which SNPs involved in skin pigmentation have allowed a better knowledge about the genetic make-up of dark skin people belonging to African and African-derived populations. 

#### 3.5.4. Results of Ancient DNA Samples 

Six ancient DNA samples were included in this study, even if after DNA extraction and quantification only two samples displayed a DNA amount suitable for library preparation (Supplementary Table S1).

The first ancient DNA sample, BAS539, was found in the Necropolis Collatina, not far from the centre of Rome and roughly dated between the 1st and the 3rd cent. CE [17]. The MPS library was prepared to amplify 130 pg of input DNA using 23 PCR cycles. The mean amplicon coverage was 833.1, but a partial profile with four no-called loci was obtained for these samples. The genotyping of 37 SNPs was uploaded to HIrisPlex-S webtool and subjects with brown eyes, dark brown/black hair and intermediate to dark skin were predicted. The four missing SNPs were related to hair (rs4959270) and skin (rs2238289, rs6119471, rs6059655) predictions and produced an AUC loss ranging between 0.001–0.002 for hair prediction and 0.001–0.006 for skin prediction (Supplementary Table S4). The phenotypic prediction copes well with the bioarchaeological interpretation of the burial ground. Indeed, the Necropolis Collatina is one of the largest Roman burial grounds to date, and it is thought to host people from the Suburbium, which was populated by individuals of Eastern Mediterranean and North African genetic origin [22,23,42].

The second archaeological sample, FIDENE, belongs to a hydrocephalic child from a 1995 excavation of a cemetery close to suburban estates close to the ancient town of Fidenae and is dated between the end of the first century and the beginning of the second century DC. The MPS library was prepared by amplifying 55 pg of input DNA using 23 PCR cycles. The mean amplicon coverage was 247.6 and partial profile with five no called loci was obtained for this samples.

After MPS sequencing, the sample presented a partial profile with 36/41 SNPs genotyped, the five missing SNPs (rs3114908, rs17128291, rs1129038, rs1667394, rs1545397) were related only to skin category prediction. Despite these missing loci, intermediate skin colour was predicted for this sample, with an AUC loss of 0.014 (Supplementary Table S4). An inconclusive result for eye colour prediction was obtained, as probabilities for each phenotype were lower than the interpretation threshold of 0.7). Furthermore, for this sample brown/dark-brown hair was predicted by webtool. To date, the ongoing Whole-Genome Analysis is trying to dissect the genetic ancestry of the individual, even though the proximity with the Fidenae area could be meaningful for biological proximity with central Italian populations.

#### 3.5.5. Results of Casework Samples

We collected four casework samples, whose identity was already known together with their appearance. The DNA amount used for library preparation ranged between 53 pg to 338 pg and only one sample showed a complete SNPs profile. This sample, named 6916, belonged to a female subject found 18 months after its disappearance in the Po River (Italy) in an advanced stage of decomposition. The designed panel was able to predict a subject with blue eyes, and dark blond/brown hair; however, an inconclusive result was obtained for the skin category because a *p*-value < 0.5 was observed for each phenotype category. 

The remaining three samples displayed partial profiles. The 5880 sample, belonging to a missing woman found after 5 months on the bottom of Lake Garda, showed a partial profile in which the genotypes of two polymorphisms, rs4959270 on gene *EXOC2* and rs6119471 on gene *ASIP* were lost. These two missing SNPs affect the hair and skin prediction category; however, the resulting AUC loss was irrelevant for the determination of phenotypic categories (ranging between 0.001–0.002 for the hair category and 0.001 for the skin category). The phenotype predicted agreed with the description of the woman as a subject with brown eyes, and dark brown/black hair, but also for this sample an inconclusive result for skin phenotype was obtained. 

We observed an unexpected result for sample 5672, belonging to a man found 17 days after his disappearance, whose semi-carbonized corpse was buried in the countryside. For this sample, only a partial profile was obtained, with nineteen SNPs typed despite the library being prepared with 338 pg of input DNA. Moreover, library concentration was low (5.095 pM) and final MPS results showed a low mean depth value of 192.8 and only 23% of the reads on target. The previous STR typing performed on the same DNA sample showed a partial STR profile, displaying a gently slope down, suggesting a mild degradation status for this DNA sample. As the SNPs panel in this study was designed with amplicons sized below 174 bp, the DNA degradation does not seem to be the cause of the partial SNPs profile. Therefore, some not well-identified problems during library preparation were hypothesized for this sample. No predictions for eye colour were obtained because missing the input for rs12913832 (*HERC2* gene) as described in [46]. Similarly, no hair colour was predicted because no genotype for SNPs rs12913832 (*HERC2* gene), rs16891982 (*SLC45A2*) and rs12203592 (*IRF4*) was detected as described in [46]; furthermore, an inconclusive result was obtained for skin category. 

The last casework sample belongs to a young 15-year-old woman of Bengali descent whose bones were found eight years after her disappearance. This sample showed a very low DNA concentration (0.008 ng/µL) and therefore the maximum DNA amount available for library amplification was 53 pg. As our sensitivity studies clearly showed an increase in allelic drop-out when using less than 100 pg of input DNA. We performed two replicates of library and MPS, in order to consolidate the resulting genotypes. The consensus profile consists of 40/41 SNPs, with no-call at rs6119471 (*ASIP* gene) that affects the skin prediction category and produced an AUC loss of 0.001. The resulting phenotypes prediction for this sample were brown eyes, black hair and dark skin. Remarkably, for all these samples the predicted phenotypes fully agreed with the expected ones. 

#### 3.5.6. Results of Touch DNA with Reference Samples

The last samples collected for this study were five touch DNA samples, together with their reference buccal swabs. The availability of the reference samples allowed us to perform a more reliable analysis on these challenging samples. It is well-known that touch DNA often contains not only a very low DNA amount, but it is more prone to suffer from DNA contamination (e.g., background DNA), which would compromise the genetic appearance prediction results. The touch DNA samples showed a DNA concentration ranging from 0.075 ng/µL to 5.487 ng/µL with a degradation index ranging between 0.70 and 2.3 (Supplementary Table S1); in addition, the qPCR quantification assay suggested the presence of PCR inhibitors in samples T001, TO18 and TO24. Libraries preparation for the buccal swab samples was performed in half-volume reagents. The concentrations of the half-volume libraries were similar to the concentration of the libraries performed with the same DNA input (see the section on sensitivity study), but with the recommended reagents volumes. The MPS libraries showed a concentration between 32.790 pM to 199.683 pM (Supplementary Table S2).

All the samples showed a full profile with concordant genotyping and phenotyping results, except for the reference buccal swab TO06. Despite the DNA input used for the amplification being 2.1 ng and the library quantification being comparable to that of other successfully sequenced samples, after sequencing only 11.5% of reads on target with 43.6% uniformity were found. These results suggest an imbalance between the two primer pools used for amplification. The sample showed a partial profile with 30/41 SNPs correct genotyping, these missing polymorphisms affect all prediction categories with AUC loss ranging between 0.010–0.040 for eye prediction, 0.004–0.019 for hair prediction, 0.005 for shade prediction and 0.005–0.016 for skin prediction (Supplementary Table S4). Nonetheless, the predicted phenotypes for TO05 and TO06 are concordant and showed a subject with brown eyes, dark brown/black hair and intermediate skin.

Interestingly, the samples TO17 and TO18 showed hair darkening during childhood with an equal *p*-value for the blond and brown categories, 0.416 and 0.452, respectively [48]. 

## 4. Concluding Remarks

This study aims to develop a multiplex MPS assay to genotype all the 41 SNPs included in the HIrisPlex-S system for simultaneous prediction of eye, hair and skin colour. Several types of challenging forensic samples, different for biological matrix and degradation status, were used to evaluate the performance of the MPS-designed panel. The panel was evaluated by testing different numbers of PCR cycles (21, 23 and 25) and reagent volumes used in the PCR for library building (full or half-volume). Note that the use of half volumes for the PCR and library preparation did not compromise the quality of sequencing results, which is comparable to that of libraries prepared according to recommended volumes, amplifying the same amount of DNA.

The MPS assay showed a very good performance in terms of sequence quality and coverage. The 41 loci have been uniformly amplified and sequenced in all different types of samples, without any difference between reference and degraded DNAs and regardless of the amplicon’s size. From the analysis of rDoc one amplicon, AMPL7160226302 with rs6119471 displayed a remarkable increase in rDoC value both in amplified reference samples with low input DNA and in degraded samples. After an in-depth review by IGV software, we considered that the high number of reads found is due to a high number of dimers that are formed during the amplification reaction, given the quality and quantity of DNA for these samples. Furthermore, though this locus is repeatedly flagged (COV) in several samples, IGV inspection confirms the genotyping obtained by the plugin in all samples.

As assessed by sensitivity test, reliable genotype profiles were observed up to 100 pg DNA input and with 23 PCR cycles. Drop-out started to occur at 50 pg and at 25 pg input DNA, more drops out events occurred for the amplified samples at 23 and 25 PCR cycles. It is interesting to note that the increment of the number of PCR cycles results in improvement of sensitivity or in typing results, but an increase in artefacts was also observed. 

The phenotyping prediction was obtained using the HIrisPlex-S webtool (https://hirisplex.erasmusmc.nl/ (accessed on 15 April 2022)). Eye and hair colour was predicted in 92.60% of individuals and skin colour in 85.15% of individuals. The most frequently missing genotypes were observed for the rs6119471 on ASIP gene, which has an impact on skin colour prediction category for differentiation between African versus non-African. Interpretation of the results of eye, hair and skin colour obtained by the HIrisPlex-S Webtool was performed using the prediction guide recommendations. Brown eye colour was predicted in the majority of cases (16, 62.5%), blue in eight samples (33%); however, inconclusive prediction (probabilities for each phenotype <0.7) was noted in one sample. For hair colour prediction, the majority of samples showed a dark brown–black colour (52%), followed by blond or dark blond category (28%). In one single sample, red hair colour (4%) was predicted, as well as dark blond/brown and dark brown/brown categories. Interestingly, for one individual we noted a hair darkening during childhood, with equal *p*-value for the blond and brown category, 0.416 and 0.452, respectively. The skin colour prediction algorithm predicted most individuals (18, 78.26%) to fall between II, III and II/III (pale to intermediate category) on Fitzpatrick’s Scale. Four individuals (22.22%) belonged to V Fitzpatrick’s category (dark to Black skin prediction). Finally, three samples showed an inconclusive result (highest prediction probability lower than <0.5) based on the skin prediction guide proposed by Chaitanya and colleges.

In conclusion, the study demonstrated the panel’s ability to process low-level or degraded DNA typically found in forensic casework. The results show that full and reliable profiles were obtained with 0.1–5 ng, even with degraded DNA. The tests performed in this study show that the use of half volumes reagent for library preparation is applicable for good quality reference samples, but nothing can be inferred for challenging casework samples, as no tests were performed on this sample’s type.

Based on our results, the DNA degradation status did not influence the correct genotyping and phenotyping and the critical parameter affecting the result is the quantity of input DNA. However, we recommend carefully setting the analytical threshold for locus calling and proceeding to a manual review of the results for samples with low DNA quantity and/or quality, preferring, where possible, a replication of the samples in order to avoid data loss.

## Data Availability

Data is contained within the article or supplementary material. For information about the designed MPS panel please contact the corresponding author.

## Supplementary Materials

The following supporting information can be downloaded at: https://www.mdpi.com/article/10.3390/genes13101688/s1, Table S1: List of the samples analysed in the present study. The biological and molecular features of the samples, the DNA extraction protocols used, the DNA concentration and degradation index (for samples quantified with Quantifiler™ Trio DNA Quantification Kit the Small Autosomal (SA) target values are reported); Table S2: MPS libraries list submitted to analysis in the present study. The libraries were pooled based on reagent volume for library building (full or half volume). In the first column was reported the sample followed by the amount DNA input used for amplification, the PCR cycles and the results quantification of libraries. In the following columns were reported the Ion Torrent technology, chip type and sequencing session. In the remain columns were summarized the information of the Coverage Analysis plugin.; Table S3: Main parameters of chip use in the present study; Table S4: Phenotype predictions obtained by HIrisPlex-S web tool. In the first column was reported the sample name, the genotype profile obtained for each samples followed by the number of missing SNPs. In the remain columns were reported the expected phenotype, subsequently the predicted color category for each samples together to *p*-value of predicted category and AUC loss value, which expresses a decrease in model prediction accuracy in the lack of genetic information.
